# Human body temperature cues widespread changes in virulence gene expression in uropathogenic *Escherichia coli*

**DOI:** 10.1128/iai.00422-25

**Published:** 2025-12-23

**Authors:** Carolyn A. Dehner, Isidora N. Stankovic, Madeleine Sutherland, Lou Ann Bierwert, Kalina P. Dimova, Stylianos P. Scordilis, Daniel M. Stoebel, Christine A. White-Ziegler

**Affiliations:** 1 Department of Biological Sciences and Program in Biochemistry, Smith College 6089, Northampton, Massachusetts, USA; 2 Department of Biology, Harvey Mudd College, Claremont, California, USA; Tsinghua University, Beijing, China

**Keywords:** temperature, virulence, immune evasion, RpoS, stress response, biofilm, transcriptional regulation, transcriptome, proteome, sRNA, fimbriae, adhesion

## Abstract

As a bacterial pathogen enters a human host, it immediately encounters a temperature upshift to 37°C. Mimicking the early hours of infection, we analyzed the transcriptome and proteome of uropathogenic *Escherichia coli* CFT073 initially grown at 23°C, then shifted to 37°C for 4 h. Temperature caused a change in mRNA expression for 9% of the genome (1% false discovery rate, ≥2-fold); similar impacts were observed for the proteome with a good concordance amongst the most highly temperature-regulated genes. Comparison to *E. coli* K-12 MC4100 shows temperature to be a more broadly used regulatory cue in the uropathogen. Multiple operons associated with fimbrial adhesion, biofilm formation, immune evasion, and competitor defense show temperature regulation. Multiple fimbrial adhesins (*pap*, *pap-2*, *foc*) are increased in expression at 37°C, while others (*ecp*) are favored at 23°C. Decreased motility gene expression at 37°C and 23°C is correlated with the thermoregulation of multiple motility repressors (*papX*, *focX*, *pdeL,* and *rpoS*). Several biofilm formation and c-di-GMP signaling genes showed preferential expression at 37°C, suggesting human body temperature modulates this process. Growth at 37°C promotes a broad set of immune evasion genes (complement evasion, antimicrobial peptide cleavage, phagocyte killing/iron acquisition, copper export) along with genes associated with competitor bacterial and phage defense. RpoS protein expression and the genes it controls show minimal changes during this time course, indicating bacteria enter the host ready to counter diverse stresses in various niches. Together, our studies demonstrate that temperature cues a suite of genes whose expression benefits host colonization and survival.

## INTRODUCTION

Uropathogenic *Escherichia coli* (UPEC) are the cause of 70%–95% of community-acquired urinary tract infections (UTIs) and nearly one in two females will experience a UTI in their lifetime (1, 2). UPEC can cause infection of the bladder (cystitis) or a more serious ascending infection of the kidney (pyelonephritis). UTIs account for approximately 50% of hospital-acquired infections in the United States (2) with the most common being catheter-associated UTI caused by UPEC that forms biofilms on these indwelling devices, frequently leading to bacteriuria (1). In the United States, increasing office and emergency visits along with increased hospitalizations are associated with 2.8 billion in annual hospital costs that coincide with an increase in antimicrobial resistance (3).

Bacteria encounter vastly different environments during their life cycle; therefore, they must sense and adapt to changing conditions quickly to ensure survival. Using temperature as a cue to discriminate between external and host environments and subsequently regulate gene expression has been documented in a number of different genera of bacteria, at both the operon and genome-wide level. Early thermoregulatory investigations often focused on virulence determinants required for colonization and adhesion, but genome-wide studies have revealed a broader array of genes that are impacted by temperature changes including stress responses, central metabolism, nutrient acquisition, and respiration strategies (4–11).

A temperature shift from 23°C to 37°C that mimics the transition from external settings into the human host was the emphasis of our previous transcriptome study in the commensal *E. coli* K-12 MC4100 strain. In those studies, we demonstrated that a rapid reprogramming of a multitude of responses occurs within the first few hours of a temperature transition. The first hour at 37°C is characterized by a transient shift to anaerobic respiration strategies and stress responses, particularly acid resistance, indicating that temperature serves as a sentinel cue to predict and prepare for various niches within the host. By 4 h, gene expression patterns shifted to aerobic respiration pathways and decreased stress responses, coupled with increases in genes associated with biosynthesis (amino acid, nucleotides), iron uptake, host defense, and motility (12). Many of these responses initiated in the first few hours are retained for long-term growth at 23°C or 37°C (10, 11).

Our genome-wide studies in non-pathogenic *E. coli* K-12 MC4100 support the model of RpoS as the primary thermoregulator of stress response gene expression. Thermoregulation of RpoS expression, mediated by the small regulatory RNA (sRNA) DsrA that facilitates *rpoS* mRNA translation at low temperature, had been previously characterized with its impact demonstrated for a limited set of genes (13, 14). Our results in *E. coli* K-12 MC4100 confirmed this temperature-mediated regulation for a larger subset of RpoS-dependent genes (11) and implicated another sRNA RprA in RpoS thermoregulation (12). While a temperature upshift in *E. coli* K-12 MC4100 mediates an eventual decrease in RpoS expression, our results demonstrated that a microbe at 23°C would enter the host with high levels of RpoS that could sustain stress resistance. While RpoS can account for the thermoregulatory control of a significant number of genes, the mechanisms for the remaining temperature-controlled genes are yet to be determined.

In this study, we examined how the temperature upshift from 23°C to 37°C impacts the uropathogen *E. coli* CFT073. In this novel study using both transcriptomic and proteomic strategies, our findings reveal a wealth of genes whose expression is increased by this temperature upshift that promote host colonization via adhesion, immune evasion, and biofilm formation with the concomitant retention of a defensive robust stress response.

## RESULTS

### Temperature broadly impacts mRNA and protein expression in uropathogenic *E. coli* CFT073

**Fig 1:**
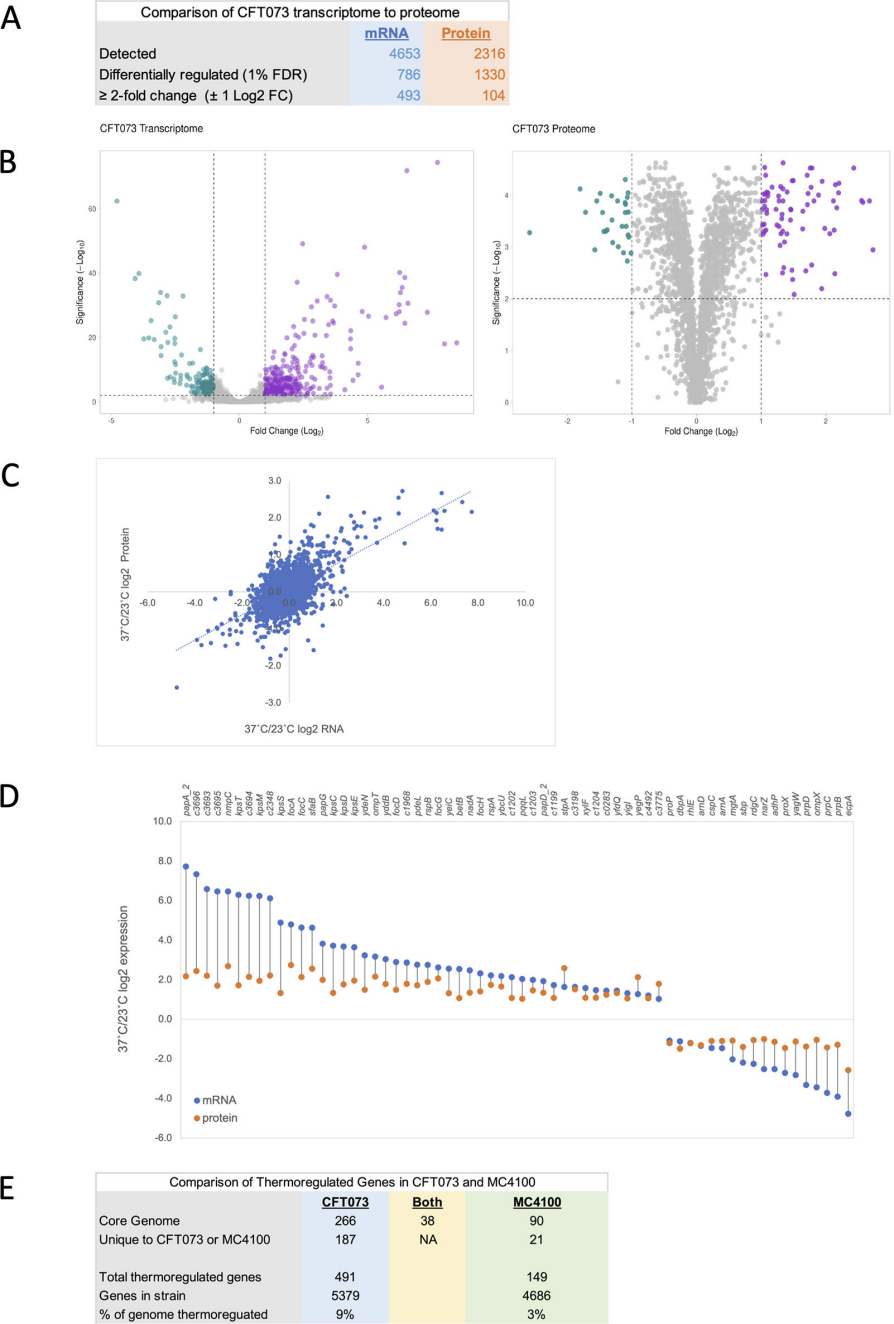
Characterization and comparison of the transcriptome and proteome of uropathogen *E. coli* CFT073 4 h after a shift from 23°C to 37°C. (**A**) Comparison of the differentially regulated genes and proteins 4 h after the temperature upshift from 23°C to 37°C. (**B**) Volcano plots of the transcriptome and proteome with each dot representing a unique mRNA/protein. mRNAs/proteins with significantly increased expression (magenta) or decreased expression (green) (FDR ≤ 1%, at least ±1 log2FC) are indicated. Volcano plots were created using VolcaNoseR (15). (**C**) For genes detected in both the transcriptome and proteome, the 37°C/23°C log2FC of each mRNA is plotted on the *x*-axis in comparison to its 37°C/23°C log2FC protein on the *y*-axis. (**D**) Comparison of 37°C/23°C log2FC for mRNA (blue dot) to protein (orange dot) for those genes with FDR ≤ 1% and at least ±1 log2FC in both the transcriptome and proteome. (**E**) Comparison of differentially regulated genes in *E. coli* CFT073 to genes in *E. coli* MC4100.

Mimicking the transition experienced by a bacterium as it moves from the external environment and enters a human host, this study aimed to identify and characterize the suite of genes whose expression changes upon a temperature upshift from 23°C to 37°C. The temperature-dependent experimental growth conditions were designed to ensure that cultures were initiated and harvested in early-mid exponential phase (A_600_ = 0.3–0.6) for all time points, allowing gene expression changes to be directly attributed to the temperature shift and not to changes in nutrient levels or growth phase. RNA-Seq analyses were completed by comparing expression at t = 4 h after the shift to 37°C with cells retained at 23°C. RNA was detected for 86% of the genome (4,653 genes, Table S1). Using a false discovery rate (FDR) of ≤1%, 786 annotated genes showed statistically significant gene expression changes (Table S2); in this study, the analyses were focused on the 493 annotated genes with FDR ≤ 1% whose expression was altered by at least ± 1 log2FC (log2 fold change), representing approximately 9% of the genome (Fig. 1A and B; Table S3).

In samples grown under identical conditions for proteomic analyses, an overall comparison revealed that protein differential expression ratios were compressed as compared to mRNA, but predominantly showed the same thermoregulatory trends (Fig. 1C and D). Of the 2,316 proteins detected (Table S4), the temperature upshift led to the differential expression of 1,330 proteins (FDR ≤ 1%, Table S5), of which 104 displayed a differential of at least ±1 log2FC (Fig. 1A; Table S6).

Strictly limiting a comparison of proteins to genes with at least an FDR ≤ 1% and at least a ± 1 log2FC in both data sets revealed an overlapping subset of genes that were temperature regulated at both the mRNA and protein levels. These are primarily aligned with some of the most highly transcribed genes based upon RPKM (reads per kilobase per million mapped reads) values (Fig. 1D; Table S7). Many of these genes are linked to pathogenesis and host survival (e.g., fimbriae, capsule, immune evasion, and outer membrane proteins) and are highlighted in subsequent sections of this paper. A small protein subset (37 proteins) demonstrated altered expression (at least ± 1 log2FC), in the absence of significant mRNA changes, that could indicate post-translational mechanisms of control. Notably, several proteins associated with cold shock and growth at low temperature (CspI, YdfK, RNA DeaD helicase, gamma-glutamyltransferase, Bluf/BluR) show decreased protein levels upon the temperature upshift that were not reflected in the transcript levels (11, 16, 17).

### Temperature regulation of gene expression is not highly conserved with *E. coli* K-12 MC4100

Comparison of the differentially regulated genes in this study to results from our similar transcriptome study in *E. coli* K-12 MC4100 (12) showed little commonality in the thermoregulation of the shared core genome between the two strains with only 38 genes demonstrating temperature regulation in both transcriptomes (Fig. 1E; Table S8). More genes within CFT073 are thermoregulated, both genes within the *E. coli* core genome and genes unique to CFT073. Together, these data suggest temperature regulation of gene expression is a more broadly used regulatory mechanism in pathogenic CFT073 than in the non-pathogenic *E. coli* K-12 MC4100 and that the thermoregulation of the core genome is not highly conserved.

### Adhesion is favored at 37°C and 23°C, but through different fimbrial adhesins

**Fig 2:**
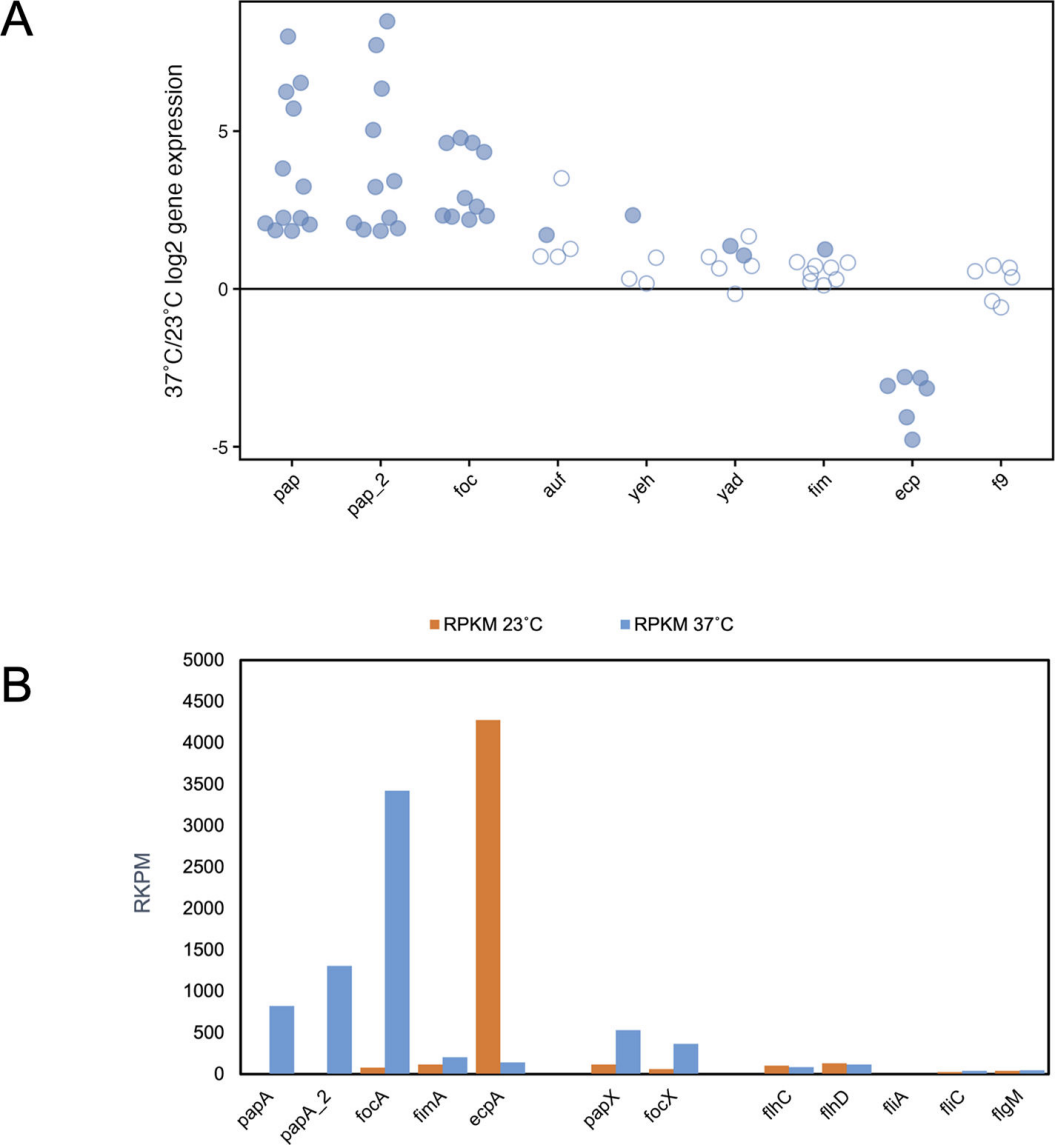
Temperature impacts on fimbrial adhesion and motility gene expression. (**A**) 37°C/23°C log2FC for each gene in a given fimbrial operon is plotted on the *y*-axis. Filled circles indicate statistically significant differential expression (FDR ≤ 1%). Data for panel A are detailed in Table S9. (**B**) The RPKM for various fimbrial major subunits and their regulators, along with motility regulators and the flagellar major subunit, are shown at 37°C (blue) and 23°C (orange).

A prominent virulence feature of CFT073 is its repertoire of fimbrial adhesins, which allow it to bind to epithelial cells, establish itself within various niches of the body, invade host cells, and form biofilms. In this experiment, transcription of the two P fimbrial operons (*pap* and *pap-2*) and the F1C operon (*foc*), all implicated in UTIs (18–21), showed the greatest temperature differential (Fig. 2A; Table S3). An analysis of the RPKM data of the fimbrial subunits indicates that these three operons are highly transcribed at 37°C (Fig. 2B) and the proteomics data identify fimbrial proteins from the *pap*, *pap-2*, and *foc* operons as some of the most highly differentially expressed proteins upon the temperature upshift (Fig. 1D; Table S3), aligning with the transcript data. Thermoregulation of *pap* gene expression has been documented (22–25); whereas, to our knowledge, this is the first observation for the *foc* operon. Genes within fimbrial operons associated with biofilm formation and/or cellular adherence—*auf* (c4209-c4214), *yad* (c0166-c0172), and *yeh* (c2635-c2638)—showed higher expression at human body temperature (Fig. 2A; Table S3), but their expression, based on RPKM (data not shown), is substantially lower than the other fimbrial operons at either temperature (26–28) and they were not detected in the proteome. Contrary to studies in *E. coli* K-12 MC4100 (29, 30), the majority of genes within the type I pili (*fim*) operon showed no thermoregulation in this study, low expression based on RPKM (Fig. 2A and B), and only FimB was detected in the proteome (Table S3).

At both the mRNA and protein level, the *ecp*/*mat* genes show the opposite thermoregulation with substantially higher expression at 23°C than at 37°C (Fig. 2A; Table S3) and are some of the most highly expressed genes at 23°C based on RPKM data and proteomics data (Fig. 1D, 2A and B; Table S3). These results confirm the temperature regulation previously reported for the *ecp/mat* genes (31, 32). The ability of ECP to foster biofilm development (31, 33, 34) and adhesion to plant glycans may implicate important roles for these fimbriae outside a mammalian host (35). This temperature regulation seems curious in light of other studies in urinary and diarrheagenic strains of *E. coli* where ECP has been shown to play a role in epithelial cell colonization and infection; however, studies have indicated that other host cues may be vital to *ecp in vivo* expression at 37°C (32, 36–38). In contrast to previous studies (39), expression of F9 fimbriae, implicated in abiotic biofilm formation in CFT073 (40) and binding of plant glycans (41), showed no thermoregulation and low expression in this study.

### RpoS and fimbrial crosstalk impact the temperature regulation of genes involved in motility

While temperature regulates fimbrial adhesins, no temperature regulation of 41 motility genes [*flh* (5), *fli* (20), *flg* (14), and *mot* (2) genes] is observed in CFT073 and further investigation of RPKM data indicates low transcript levels of the majority of motility genes at both temperatures, including the master regulator *flhDC*, the flagellar sigma factor *fliA,* and the flagellin subunit *fliC* (Fig. 2B; Table S1). These data correlate with the proteomics data where FliC and FlgM, the anti-sigma factor to FliA that prevents it from binding to sigma 28 promoters, were the only flagellar proteins detected (Table S1). This distinctly contrasts with *E. coli* K-12 MC4100, where we demonstrated that motility is increased upon a temperature upshift (12). Interestingly, it does concur with a murine infection model where motility gene expression was found to be decreased *in vivo* for *E. coli* CFT073 (42).

**Fig 3:**
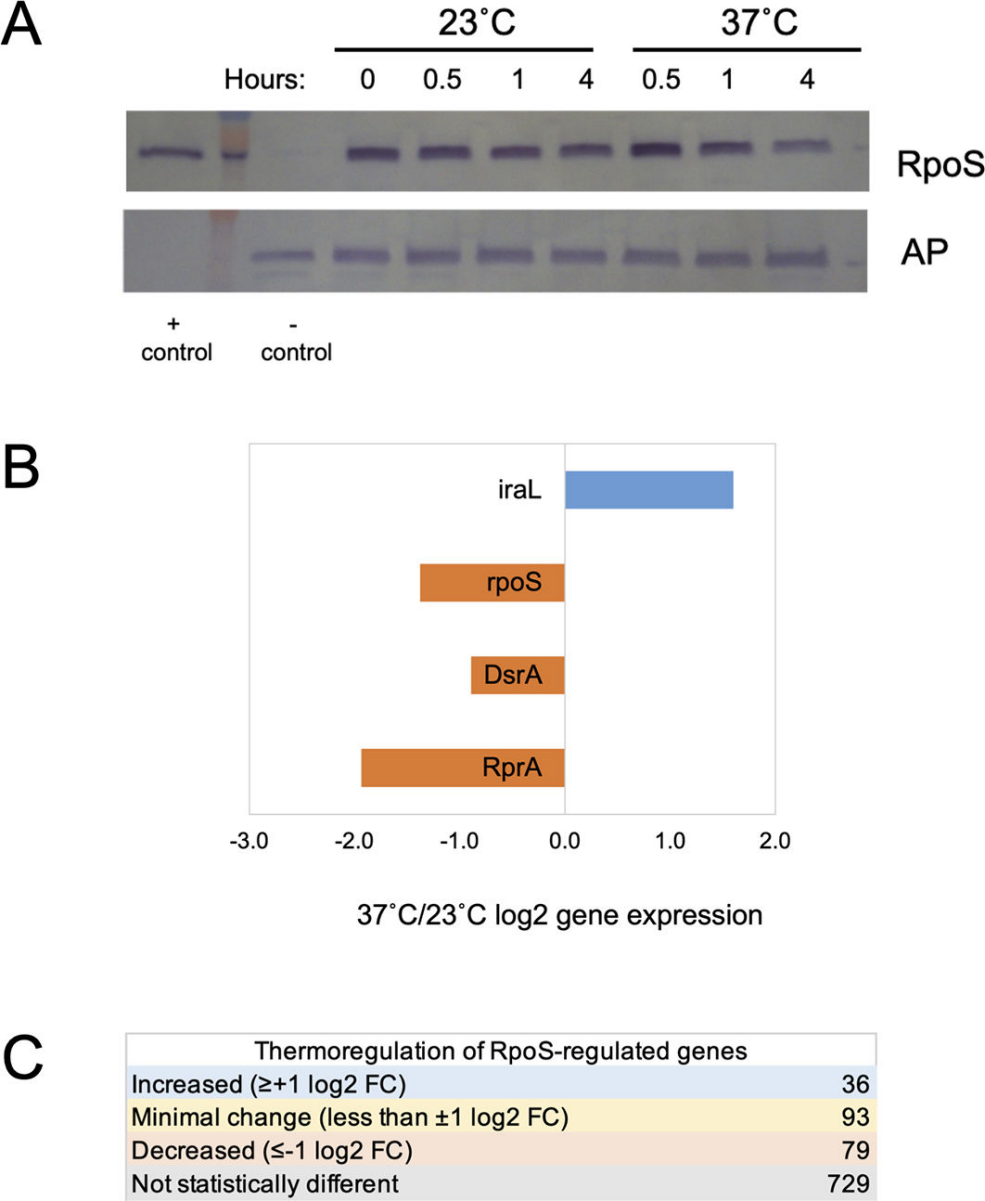
Impacts of a shift to 37°C on RpoS protein levels, regulators of RpoS expression, and RpoS-regulated genes. (**A**) Western blotting of RpoS protein in cultures retained at 23°C and those shifted to 37°C. + control = purified RpoS protein, − control = total protein from ∆*rpoS* strain. Alkaline phosphatase (AP) was used as a non-thermoregulated control. A representative experiment is shown. (**B**) 37°C/23°C log2FC mRNA of *rpoS* and the regulators of RpoS stability (*iraL*) and RpoS translation (DsrA, RprA). (**C**) Analysis of CFT073 thermoregulated genes that are RpoS-dependent in *E. coli* K-12 MC4100.

We hypothesize that RpoS may contribute to decreased motility at 23°C based on its role in *E. coli* K-12 strains where RpoS serves as a repressor of motility (43, 44). RpoS impacts motility through various mechanisms: direct competition of RpoS with other sigma factors for the RNA polymerase enzymatic core and the action of RpoS-dependent regulators (CsgD and CpxR) that decrease both flagellar gene expression and motion through repressing *fliA* transcription (45, 46). Our data demonstrate that RpoS is present at 23°C throughout the time course of this experiment (Fig. 3A) and could be contributing to the lack of motility gene expression observed.

At 37°C, we hypothesize that fimbrial crosstalk may contribute to low motility gene expression at 37°C as the motility inhibitors *papX* and *focX*, located within their respective fimbrial operons, are increased upon the temperature upshift (*papX* = 1.9 log2FC, *focX* = 2.2 log2FC) (Fig. 2A; Table S3). These regulators repress transcription of *flhD,* a subunit of the master controller of flagellar gene expression, and thus serve to coordinate crosstalk such that fimbrial and motility gene expression do not occur simultaneously (47, 48). Together, our data indicate that (i) both temperatures increase the expression of adhesive molecules with each temperature impacting different fimbrial operons and (ii) that temperature-regulated increases in fimbrial gene expression are correlated with a lack of flagellar gene expression at both temperatures.

### RpoS expression is retained upon a temperature upshift, fostering stress resistance gene expression

Beyond motility, RpoS is most well characterized in *E. coli* K-12 strains for its role in regulating stress responses (reviewed in reference 49). In *E. coli* K-12 strains, RpoS expression is favored at low temperature, mediated by temperature-regulated small RNAs DsrA and RprA, whose increased transcription at 23°C leads to increased translation of RpoS and a subsequent activation of a suite of stress response genes (11, 12, 14).

Highly similar to what we observed in *E. coli* K-12 MC4100 (12), RpoS protein expression in CFT073 is retained in cells upon a temperature upshift and only begins to dissipate 4 h after the upshift to 37°C (Fig. 3A). The visible reduction of RpoS after the shift is independently confirmed in the proteomics analysis with a statistically significant, 30% reduction in RpoS (C_RS15710/c3307) at 37°C as compared to 23°C at 4 h (Table S5).

Multiple factors may contribute to the retention of RpoS levels at levels similar to the 23°C starting culture upon the initial upshift to 37°C, followed by its subsequent decrease at 4 h. In *E. coli* K-12, a heat shock leads to the initial stabilization and increased half-life of RpoS through decreased proteolysis (12, 50). In addition, our RNA-Seq results show a 1.6 log2FC increase in *iraL* (c1429) expression; *iraL* encodes an RssB anti-adaptor that has been shown to reduce proteolysis of RpoS and leads to sustained RpoS stability in exponential phase (51). These stabilizing influences during the upshift are likely counterbalanced by decreased expression of the *rpoS* mRNA and the regulatory RNAs that foster RpoS translation at 23°C in *E. coli* K-12 MC4100 (14, 52). Similar to our results in *E. coli* K-12 MC4100 (12), DsrA and RprA are thermoregulated with lower expression by t = 4 h after the temperature upshift (Fig. 3B).

Comparing genes whose expression changes in this study to a listing of genes that show RpoS-dependence in *E. coli* K-12 (53), only a small subset of genes in CFT073 shows the expected decrease in expression upon the upshift, whereas the vast majority of genes are not temperature regulated (Fig. 3C). These results indicate that enough RpoS remains to direct the transcription of many of the stress response genes 4 h after the temperature upshift; a similar pattern is observed at the protein level. Taken together, the eventual reduction of RpoS at 37°C suggests that its expression is temperature regulated in a manner highly similar to *E. coli* K-12 MC4100 (12) with multiple regulatory strategies that maintain its expression during the initial transition to human body temperature and that likely confers stress resistance.

### Temperature has variable effects on genes involved in biofilm formation

The impacts of increased fimbrial expression coupled with decreased flagellar expression led us to examine the impact of temperature on biofilm formation genes. Indeed, fimbriae that demonstrated temperature regulation in this study—P, F1C, Yad, and ECP—contribute to biofilm formation as evidenced by both *in vivo* or abiotic biofilm assays (27, 32, 40, 54–56).

**Fig 4:**
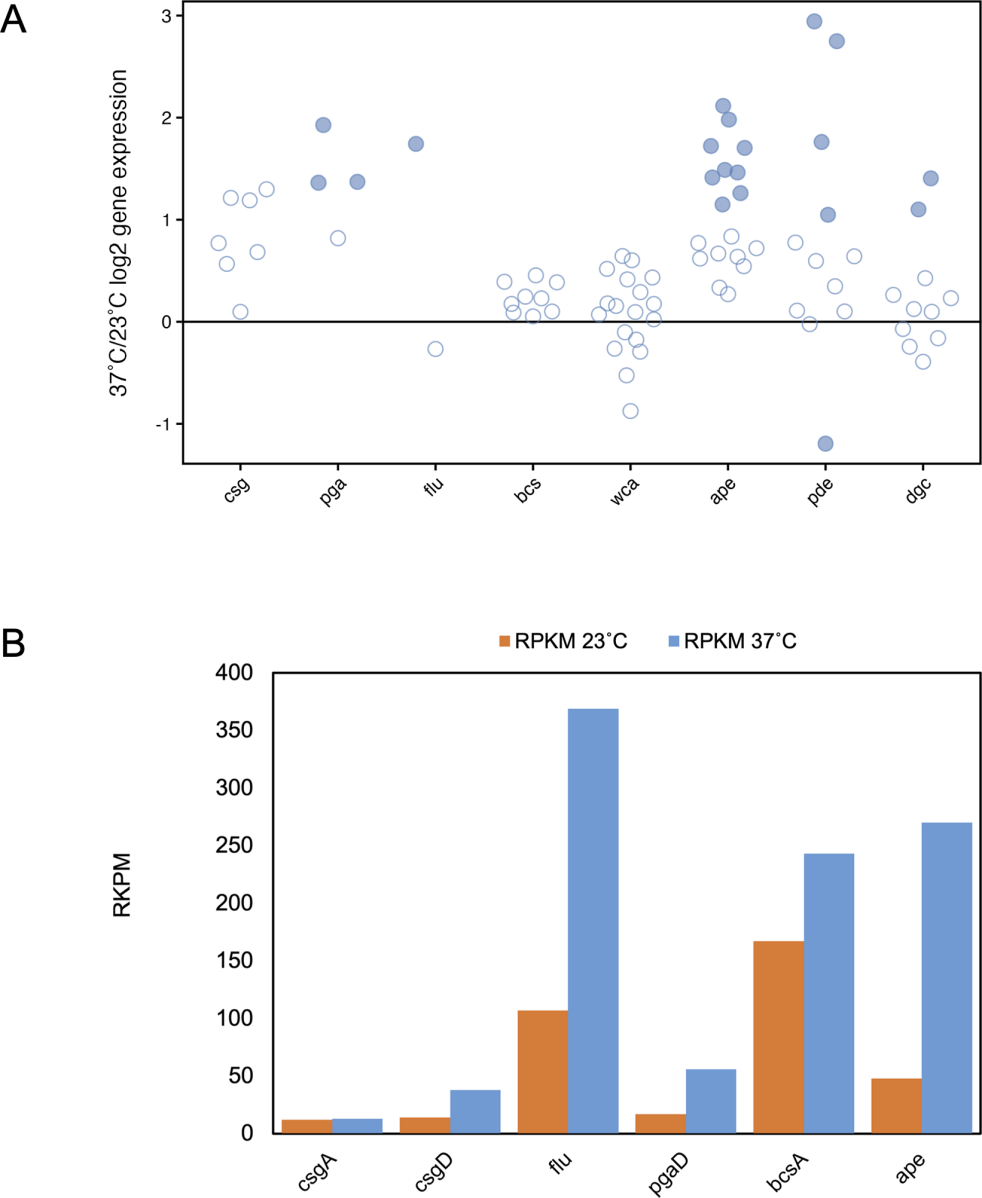
Temperature impacts on biofilm gene expression. (**A**) 37°C/23°C log2FC for each gene in operons/grouped subsets of genes related to biofilm formation is plotted on the *y*-axis. Filled circles indicate statistically significant differential expression (FDR ≤ 1%). Data for panel A are detailed in Table S10. (**B**) The RPKM for representative genes from various biofilm-related structures is shown at 37°C (blue) and 23°C (orange).

Three additional factors that facilitate biofilm formation—Ag43 (*flu,* c3655), aryl polyenes (APEs, c1186-c1204), and poly-N-acetyl glucosamine (*pga, c1160-1163*)—showed preferential gene expression at 37°C (Fig. 4; Table S3). The protein product of Ag43 and several proteins within the *ape* operon were also detected in the proteome. Ag43, prevalent in clinical urinary tract isolates (57–59), promotes autoaggregation and adhesion in intracellular UPEC biofilms and other *in vivo* infection models (reviewed in references 60, 61). Found within many host-associated bacteria, aryl polyenes confer resistance to oxidative stress and promote the formation of thicker, more voluminous biofilms in CFT073 (62). Poly-N-acetyl glucosamine (*pga*) is one of three major exopolysaccharides associated with *E. coli* biofilms (63) and increases UPEC fitness during infection (64).

Interestingly, the transcription of curli, cellulose, and colonic acid, all of which contribute to biofilm formation and are more highly expressed at 23°C in non-pathogenic *E. coli* (12, 65–68), showed no thermal regulation in this study (Fig. 4A). Curli transcription and expression in this study are neither highly transcribed nor translated at either temperature based on the RPKM and proteomics data (Fig. 4B; Table S3). Expression of *csgD* that encodes the critical RpoS-dependent activator of *csgBAC* transcription (Fig. 4B) is decreased at both temperatures, possibly contributing to low curli levels. Proteins associated with cellulose synthesis, but not colanic acid synthesis, were identified in our proteomics study.

The temperature regulation of several c-di-GMP phosphodiesterases (PDEs) and diguanylate cyclases (DGCs) that hydrolyze and synthesize, respectively, the signaling molecule c-di-GMP raises the intriguing possibility that these regulators may be impacting motility and biofilm formation. In non-pathogenic *E. coli*, high c-di-GMP levels have been associated with a decrease in motility and a transition to a biofilm state (69, 70). Three PDEs, *pdeL*, *pdeI*, and *pdeF*, and two DGCs *dgcI* (*yliF*) and *dgcP* (*yeaP*) show increased expression at 37°C whereas *pdeR* is preferentially expressed at 23°C; proteomic data confirm the differential regulation for PdeL, PdeR, and DgcP (Fig. 4A; Table S3). In *E. coli* K-12, PdeL directly represses *fli* gene expression (71) that may contribute to the decreased motility gene expression observed at 37°C in CFT073. *pdeI* was previously shown to be highly temperature regulated (72) and suppressor mutations in *pdeI* (in a ∆*pdeH* background) can lead to increased motility. Together, the differential expression of several of these enzymes, at both the transcriptional and protein level, suggests a role for temperature in controlling the motility versus sessility transition.

### Human body temperature increases expression of immune evasion and competitor defense genes

**Fig 5:**
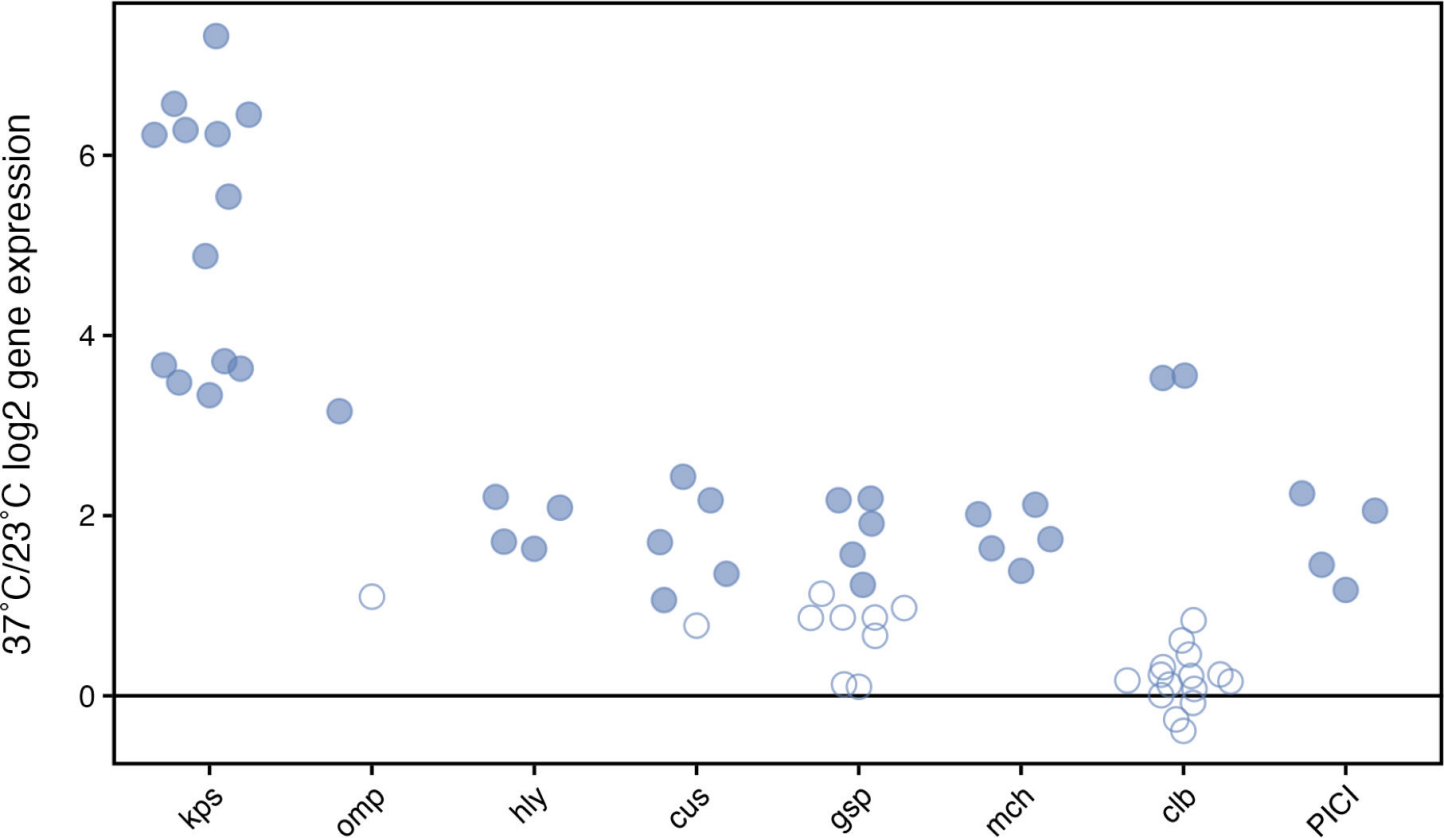
Temperature impacts on immune evasion and competitor defense gene expression. 37°C/23°C log2FC for each operon/grouped subsets of genes is plotted on the *y*-axis. Filled circles indicate statistically significant differential expression (FDR ≤ 1%). Data are detailed in Table S11.

Multiple genes that showed increased expression after the shift to 37°C—K2 capsule, hemolysin, OmpT, and OmpW—contribute specifically to evasion of host immune response. K2 capsule (*kps* and *ksl*) mediates complement evasion (both direct killing and complement-facilitated phagocytosis) (73) and has been shown to be more highly expressed during *in vivo* murine UTI (42). Genes associated with its synthesis are some of the most highly thermoregulated at both the mRNA and protein level (Fig. 1D and 5; Table S3). The pore-forming toxin hemolysin lyses many types of host cells, aiding in both immune evasion and iron acquisition from red blood cells, while at sublytic concentrations, its expression facilitates exfoliation and decreases immune signaling (reviewed in reference 74). Its thermoregulation confirms a previous study showing higher expression at human body temperature (75). We note that other toxins associated with virulence (Sat, Vac, Pic) do not exhibit temperature regulation in this study.

In both the transcriptome and proteome, the outer membrane protease OmpT is one of the most highly thermoregulated molecules (Fig. 1D and 5). OmpT cleaves antimicrobials including cathelicidin (LL-37) (76), protamine (77), bacterial colicins E2, E3, and D (78, 79), and antimicrobial proteins isolated from human urine (80). It has also been shown to participate in adhesion/invasion, IBC formation, and upregulation of host inflammatory cytokines in uropathogenic *E. coli* (81). Another outer membrane protein, OmpW, confers complement resistance through the binding of factor H (82). Along with our proteomic studies, which show protein expression at 37°C, others have confirmed the expression of OmpT and OmpW in UPEC strains grown in urine (83, 84), supporting their important role as virulence factors. Our previous studies have shown *ompT* to be temperature-regulated in *E. coli* K-12 MC4100 and enteropathogenic *E. coli* E2348/69 (12).

High expression of genes of the *cus* operon at human body temperature (Fig. 5) coordinates with *in vivo* studies showing that these genes are highly expressed in both human and mouse UTIs (85) and serve as fitness factors in mouse infection models (85, 86). The *cusCFBA* operon mediates copper efflux (87–89) that combats the toxic effects of rises in serum and urine copper levels and macrophage oxidative phagolysosomal killing (reviewed in references 85, 90).

While it is unknown what substrates it secretes in uropathogenic *E. coli*, mutations in *gsp* lead to increased bladder epithelial cell efflux and decreased kidney colonization (91), indicating a link of this type II secretion system to virulence. These data, along with studies demonstrating its transcription *in vivo* during human UTI (92), its broad conservation among many Gram-negative pathogens (93), and its thermoregulation in enteropathogenic *E. coli* (93), correlate with its increased expression triggered by host temperature (37°C) in this study.

Evasion of microbial competition is also temperature regulated as evidenced by increased expression of genes for microcin H47, colibactin, and phage-inducible genes at 37°C (Fig. 5; Table S3). Microcin H47 mimics siderophores and binds to catecholate siderophore receptors (e.g., FepA, Fiu, CirA) of competing microbes, subsequently inhibiting ATP synthase. Microcins are found in many uropathogenic strains and considered to be advantageous to colonization of the intestinal tract, a common reservoir of extraintestinal *E. coli* strain before entry into the urogenital system (94). While of high concern because of its genotoxic effects and its association with colorectal cancer (95, 96), colibactin has also been shown to be a potent bacterial weapon through its ability to induce prophage in lysogenized bacterial competitors (97). The initiators (*clbRA*) of colibactin expression show very high differential RNA levels (3.6 and 3.5 log2FC, respectively). ClbR activates transcription of other genes in the *pks* island that produce colibactin, while ClbA is the initiating enzyme for colibactin production (98). While the genes activated by ClbR do not show statistically significant temperature regulation at the transcriptional level, the self-protection protein ClbS (99) does show increased expression (1.3 log2FC), perhaps indicating a regulatory mechanism to initiate protection prior to full-scale synthesis of colibactin. Genes within the recently identified and characterized phage-inducible chromosomal island (100, 101) of CFT073 (c1498–c1501) are notably increased, raising an intriguing connection between host cues and the relationship of temperature to these unique islands whose gene products interfere with competitor phage production and facilitate horizontal gene transfer (101, 102).

### A temperature upshift affects the transcription of genes involved in osmoregulation

To protect against osmotic stress, particularly important in the changing environment of the bladder, bacteria secrete, import, or synthesize compatible solutes as osmoprotectants. The *bet* system, which synthesizes glycine betaine endogenously, shows increased expression at 37°C, confirming the thermoregulation reported in *E. coli* K-12 (103). Expression of this system protects against high urea conditions and is beneficial to growth in urine (104, 105). In contrast, the *proVWX* and *proP* operons that coordinate uptake of quaternary amines from the medium to aid in osmoregulation are significantly reduced. In *E. coli* K-12, this system is under the regulation of both osmolarity signals and RpoS (106), and their decrease may be due to decreasing RpoS levels upon the temperature upshift. Two RpoS-dependent, osmotically inducible genes with unknown function, *osmE* and *osmY*, show a similar reduction at 37°C.

### Multiple outer membrane porins are regulated by temperature

**Fig 6:**
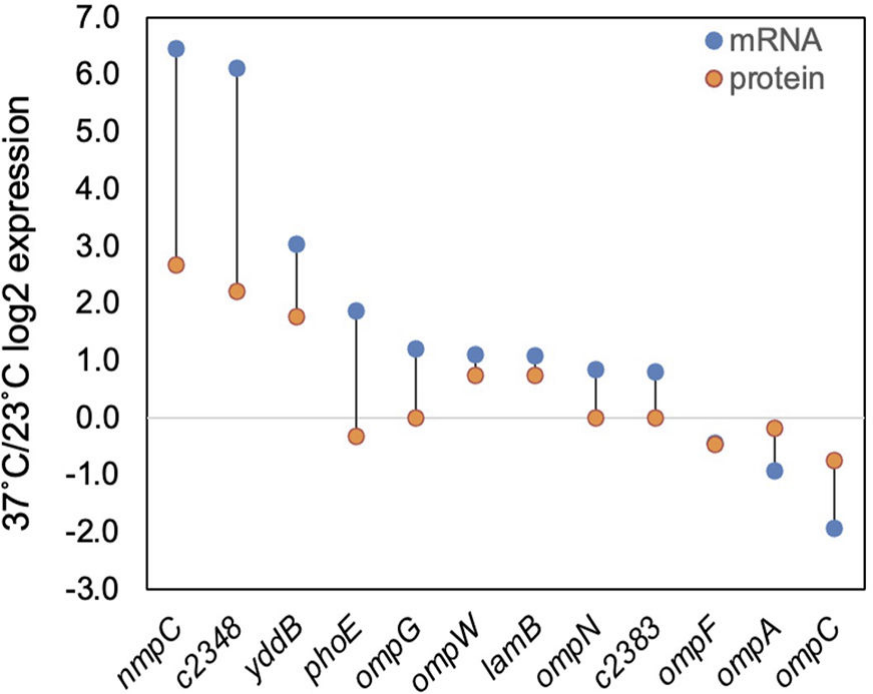
Temperature impacts on outer membrane RNA and protein expression. A direct comparison of 37°C/23°C log2FC for mRNA (blue dot) and protein (orange dot) for individual outer membrane proteins is shown.

Porins mediate movement of small molecules (e.g., sugars, amino acids, ions, antibiotics) across the outer membrane and serve as receptors for colicins and bacteriophage (reviewed in references 107–109). Additionally, they often serve as targets for host immune responses (110). Four porin genes (*nmpC/c1560*, *nmpC* precursor/*c2348*, *yddB*, and *ompG*) show preferential expression at 37°C while *ompC* is preferentially expressed at 23°C (Fig. 6; Table S3); these trends are also observed at the protein level. The well-studied thermoregulation of porins in *E. coli* K-12*,* where *ompC* expression is favored at 37°C and *ompF* is more highly expressed at 23°C (111, 112), is not conserved in CFT073.

The extremely high preferential expression of multiple less-characterized porins at 37°C is intriguing (Fig. 6). NmpC is highly expressed in the heat-resistant pathogenic *E. coli* strain AW1.7, and its overexpression has been shown to confer heat resistance (113). YddB mediates transport of a small group of antibiotics (109), is proposed to mediate iron uptake (114), and augments fitness of UPEC during systemic infection in mice (64). The thermoregulation of *nmpC* and *yddB* is conserved in *E. coli* K-12 MC4100 (12). Together, it is compelling to consider that changes in porin expression based on host temperature may predict adaptations required for the host environment and impact antibiotic resistance, given the role of porins in antibiotic transport (115).

## DISCUSSION

**Fig 7:**
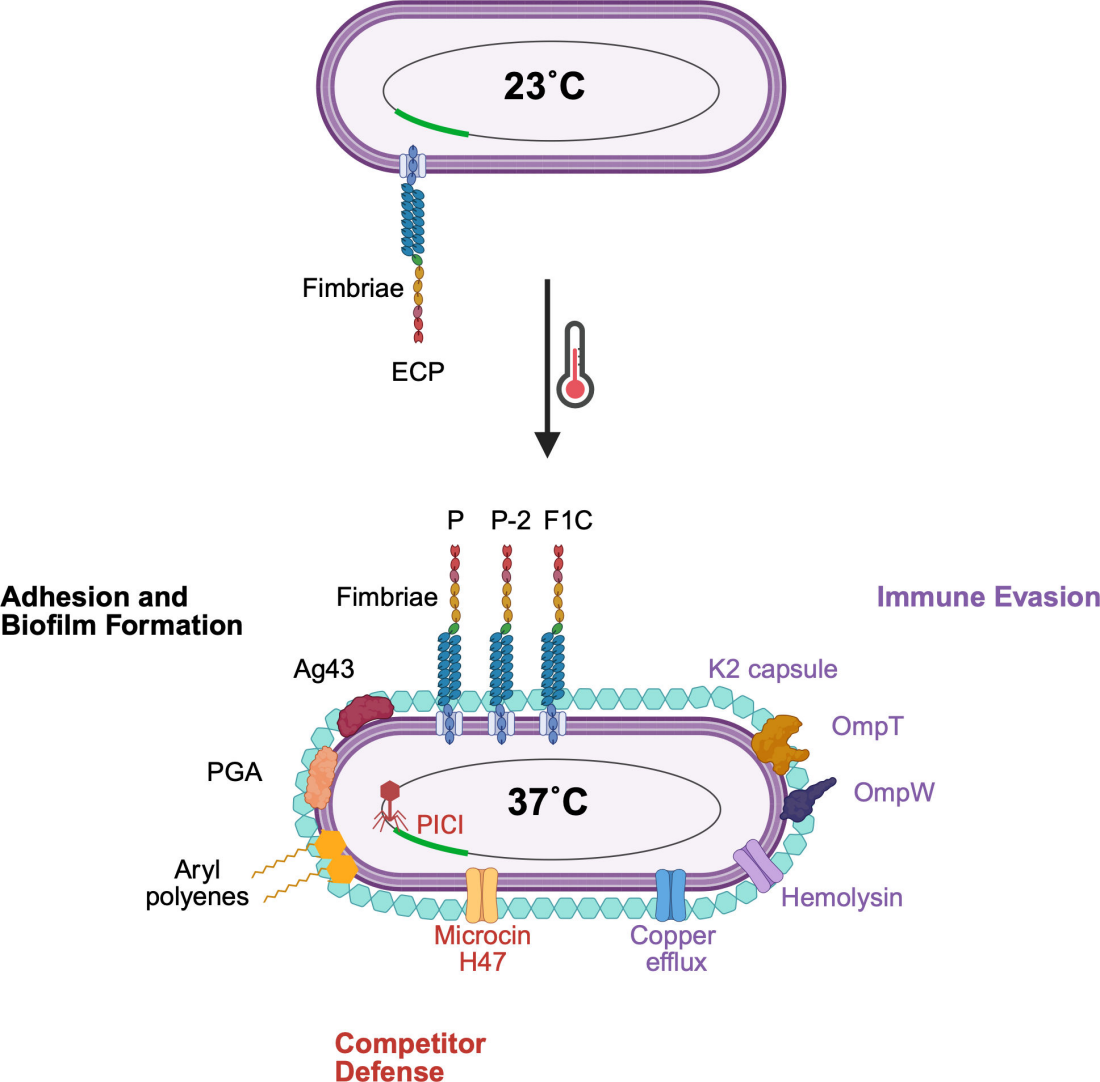
Diagram emphasizing the temperature-regulated genes in uropathogenic *E. coli* CFT073 that show differential expression after a temperature upshift from 23°C to 37°C. They are grouped by function including adhesion and biofilm formation (black), immune evasion (purple), and competitor defense (maroon). Created in https://BioRender.com.

We have demonstrated that a temperature upshift to 37°C regulates the expression of approximately 9% of the genome of uropathogenic *E. coli* CFT073, indicating it is an integral cue for programming a coordinated response to the human host environment. Many of the genes preferentially expressed at 37°C are uniquely specialized to the host, namely adhesion to epithelial cells and immune evasion through strategies that help evade complement, antimicrobial proteins, and phagocytic cell killing. Temperature also modulates genes for biofilm formation, competitor killing, and environmental stress responses that would facilitate bacterial survival and host colonization. Together, this transcriptome provides a picture of a rapidly altered set of thermoregulated responses whose expression is highly advantageous for the microbe (Fig. 7).

The paired proteome compares favorably with the transcriptome, with the vast majority of genes showing agreement between differential expression ratios at the qualitative level (i.e., increased or decreased), although the absolute differential ratios were more compressed in the proteome as compared to the transcriptome. In our review of the literature, we found few direct comparisons of bacterial transcriptomes and proteomes; ours is in agreement with previous studies that indicate a general concordance (116) and predictive value of transcriptomes for protein expression (117).

As detailed throughout this study, many of the thermoregulated genes have been shown to be expressed during growth in human urine, during infection in murine and/or human UTIs, or identified as fitness factors in various *in vivo* selections, thus correlating their increased expression at 37°C with infection (42, 64, 86, 92, 118, 119). Clearly, the evolution of sensing circuitry that links host-specific functions to temperature would be energy efficient and is observed in many pathogens (120). Defining the thermoregulatory proteins that control these genes would identify fruitful targets for novel anti-infectives that would impair the microbe’s ability to survive within the host by altering its ability to successfully adapt to the host environment.

The transition to 37°C is characterized by increased expression of genes associated with adhesion and biofilm formation, while motility genes are not highly expressed, providing evidence that temperature is an informative cue in the motility-to-biofilm transition. In this study, flagellar gene (and protein) expression is low at either temperature, while fimbrial expression is high-P and F1C fimbriae are robustly expressed at 37°C; ECP fimbriae predominate at 23°C. We should reemphasize that these results are observed in exponentially growing planktonic cells, suggesting that temperature is serving as a “priming” cue for adhesion. We posit that this transition likely occurs through the thermoregulation of key regulators—PapX, FocX, PdeL, and RpoS—that negatively impact motility. Along with high fimbrial/low motility gene expression, other various components associated with biofilm formation are either not temperature regulated or preferentially expressed at 37°C, indicating that the structural components of the biofilm formed at either temperature would differ. Together, these data suggest the biofilm state would be favored at both temperatures, perhaps helping to explain why the uropathogen is a robust biofilm former both in the host and on abiotic surfaces such as catheters.

As cells rapidly transition to the host environment, expression of the RpoS regulon decreases only minimally over the same time period, resulting in this uropathogen being stress-ready for the host environment. Upon the temperature upshift, combined factors of increased RpoS stabilization and slow diminishment of DsrA and RprA (sRNAs that facilitate RpoS translation) lead to the retention of RpoS in what would be the early hours of infection and could facilitate microbial survival as it encounters various hostile host niches. This regulation is highly similar to what is observed in *E. coli* K-12 MC4100 (12). While our results suggest that RpoS might continue to dissipate over time in our experimental system, we hypothesize that other stress-specific cues in the host (e.g., low pH, high osmolarity, oxidative stress) would sustain the RpoS-mediated stress responses.

While temperature is a cue used by *E. coli* to regulate gene expression, the lack of broad conservation of thermoregulated genes between the non-pathogenic *E. coli* K-12 MC4100 and CFT073 was a surprising result as environmental conditions (media, temperature, growth phase) were kept identical between experiments. We anticipated that the shared core genome might be regulated in a similar manner, but we found only a small subset in common between the two, with additional core (and PAI) genes thermoregulated in CFT073, but not K-12 MC4100. Perhaps this reflects a necessity to more finely tune the core genome (presumably non-virulence genes) in coordination with the numerous CFT073-specific genes that are predominantly related to infection. This lack of conservation of thermoregulation of shared genes was also observed in an analysis of multiple genera of bacteria upon a temperature downshift (121). Our ongoing studies underway in enteropathogenic *E. coli* will help to further analyze temperature regulation of genes within and among *E. coli* strains and the ways in which temperature contributes to rapid adaptation to entry into a human host.

## MATERIALS AND METHODS

### Strains and media

The strains used in this study include CFT073 (122) and CFT073 *rpoS* (WAM 2267/ATCC 700928) (123). M9 minimal (M9) and Luria-Bertani (LB Lennox) media were prepared as described previously (124, 125).

### Bacterial growth conditions

Bacterial cultures were inoculated and grown in M9 glycerol (M9 minimal liquid medium containing 2.45 μM ferric citrate, 30 μM thiamine, 100 μM calcium chloride, 1 mM magnesium sulfate, and 0.2% glycerol as a carbon source, pH 7) with aeration as described previously (10, 126). For the temperature shift, individual colonies were used to initiate a 20 mL culture. This initial culture was grown to mid-exponential phase (A_600_ = 0.2–0.6) and used to inoculate a larger, 200 mL culture that was grown to early-mid exponential phase (A_600_ = 0.2–0.6) at 23°C. This culture was diluted 1:2 or 1:3 in fresh M9 minimal glycerol medium and grown an additional 120 min at 23°C to early exponential phase (A_600_ = 0.15–0.2). At the initiation of the temperature shift, aliquots of the culture were retained at 23°C (control) or shifted to 37°C for various times (t = 0.5, 1, and 4 h). Because growth rates in M9 minimal glycerol medium differ by 2.1-fold between these two temperatures (generation time at 37°C = 1.3 ± 0.04 h; 23°C = 2.7 ± 0.04 h), the cultures at 37°C were further diluted with fresh medium at the time of the shift to 37°C to ensure that cells harvested at all time points were in early-mid exponential phase (A_600_ = 0.3–0.6).

For growth curve analyses, an initial culture was inoculated as described above and grown at 23°C to early exponential phase. The culture was subsequently shifted to 37°C, and spectrophotometer readings were taken at time points after the shift. The generation time for each strain was determined as described previously (124).

### RNA isolation

For RNA-Seq and quantitative real-time RT-PCR experiments (qRT-PCR), RNA was isolated by phenol:chloroform extraction and subjected to two DNase digestions as described previously (10). Isolated RNA was further concentrated using the RNeasy MinElute columns (Qiagen). RNA concentrations, purity, and DNA contamination levels were determined by spectrophotometer readings and Qubit assays (Qiagen). Isolated RNAs were stored at −20°C until used.

### cDNA library preparation and RNA sequencing

rRNA was depleted from 2 to 3 µg total RNA using RiboZero (Illumina). For two biological replicates, rRNA-depleted RNA was submitted to the Genome Consortium for Active Teaching for library preparation and RNA sequencing. For the remaining replicates, rRNA-depleted RNA was used for cDNA library preparation following the manufacturer’s protocol (New England Biolabs NEBNext Ultra RNA Library Prep Kit). The library was assessed for both accurate size and purity using a Bioanalyzer and sequenced on an Illumina MiSeq System in the Center for Molecular Biology at Smith College. Five paired (23°C and 37°C) independent biological replicates were sequenced.

### RNA-Seq data analysis

Residual rRNA sequences were identified and removed from the FASTQ files using an in-house script in R (unpublished). Sequence reads were mapped to the CFT073 genome using Rockhopper software (127). One 37°C sample was determined to be an outlier in comparison to the others using arrayQualityMetrics (128) and MDS plots from EdgeR (129) and subsequently removed from further analyses. Statistical testing for differential expression between temperatures was performed with DESeq2 version 1.32.0 (130). p-values were adjusted by the method of Benjamini and Hochberg (131). Genes were considered differentially expressed if adjusted p-values were ≤ 0.01 (FDR ≤ 1%) and differed by ≥2-fold (at least LFC ±1). For identification of shared genes between *E. coli* K-12 MC4100 and CFT073, EcoCyc tools (132) were used to map *E. coli* K-12 MC4100 genes to their homologous genes in CFT073.

### qRT-PCR

Reactions were completed using the SYBR Green One-Step qRT-PCR kit (Invitrogen) as described previously (10). All reactions were performed in triplicate, with no reverse transcriptase controls run for each RNA sample to detect DNA contamination. All reactions were normalized by using the same amount of total RNA (50 ng) in each reaction. Relative levels of gene expression and error analysis were calculated as previously described (10, 133).

### Protein extraction and processing for proteomic labeling

Protein was extracted from three biological replicates at each temperature (23°C and 37°C) using SDS-based lysis and chloroform-methanol precipitation as previously described (134). For quantification, 200 μg of protein extract was digested using S-Traps columns according to the manufacturer’s instructions (Protifi), then further reduced, alkylated, and trypsin-digested at 37°C overnight on the column. Hydrophilic peptides were eluted from the columns using triethylammonium bicarbonate (TEAB), and acetonitrile with 0.2% formic acid was used for elution of hydrophobic peptides. Peptide elutions from the same biological replicates were pooled and quantified (Pierce Quantitative Fluorometric Peptide Assay) before proteomic labeling.

### Peptide labeling and high-pH fractionation

Digested peptides from each sample were labeled with 10-plex Tandem Mass Tag (TMT) reagents according to the manufacturer’s instructions (TMT10plex Label Reagent, #90,111 Thermo Fisher Scientific). Briefly, 100 μg of precipitated peptides from each of the samples were dissolved in TEAB mixed with one TMT label reagent each and incubated at room temperature. After quenching, the solutions containing labeled peptides were dried using a speed-vac and resuspended in 0.1% trifluoroacetic acid solution. Before LC-MS analysis, the samples were high-pH fractionated using Pierce High pH Reversed-Phase Peptide Fractionation Kit (Thermo Fisher Scientific, #84868) according to the manufacturer’s instructions. The resulting sample eluents were then vacuum-dried and resuspended in 0.1% formic acid before LC-MS analysis.

### LC-MS analysis and protein identification

A Thermo Scientific QExactive HF-X Orbitrap mass spectrometer coupled with a Thermo Scientific Easy-nLC 1000 was used for all LC-MS analyses. Each dried peptide fraction was resuspended in 10 µL of 0.1% formic acid, and 2 µL of each fraction was desalted online on a nanoViper Acclaim PepMap 100 column and separated on a nanoViper Acclaim PepMap RSLC column (75 µm × 50 cm) at a flow rate of 300 nL/min and a 120 min gradient. Data acquisition was done using the preset TMT parameters; full MS scans of the 375–1,600 m/z range were acquired at 120 k resolution and 50 ms maximum injection time, and data-dependent MS/MS scans were acquired at 45 k resolution and 96 ms maximum injection time with collision energy set to 32 for the top 20 peaks in each full scan that met the intensity threshold of 1.0e5. The dynamic exclusion window was set to 20.0 s. The .raw files generated for all 8 fractions of each TMT comparison sample were analyzed using the Proteome Discoverer 2.4 Software (Thermo Fisher Scientific). For each study, the quantitation method was set to “TMT 10plex” and the reporter ion isotopic distribution was corrected based on the relevant product data sheet for the 10plex kit used. All searches were done using the Sequest HT database search algorithm against the UniProt database for *Escherichia coli* CFT073 (taxID: 199310 - *Escherichia coli* O6:H1). The settings allowed for full tryptic digestion, up to two missed cleavage sites, and limited to peptides of six amino acids or more. Precursor mass tolerance was set to 10 ppm and fragment mass tolerance to 0.02 Da. The Percolator algorithm was used to discriminate between correct and decoy spectrum identifications, where the maximum delta Cn for input data was 0.05. The strict target FDR was set to 0.01 and the relaxed target FDR to 0.05. Validation was based on q-values. Peptide spectrum matches (PSMs) with Sequest HT XCorr of 0.9 or higher were selected for further consensus analysis. Peptides were validated using the automatic validation mode, which controls peptide level error if possible. A strict target FDR for both PSMs and peptides was set to 0.01, and the relaxed target FDR to 0.05. Only peptides with seven amino acids or more and of high confidence were considered.

### LC-MS protein quantitation

Reporter ion quantification was based on peak integration, with a tolerance setting of 20 ppm. Both unique and razor peptides were used for reporter ion quantitation, and a peptide was only considered as shared if it referenced proteins from different protein groups. Reporter abundance was based on signal-to-noise (S/N) values with an average reporter S/N threshold of 10. Co-isolation of up to 30% was tolerated. Peptide quantitation was normalized to total protein amount: the total sum of the abundance values for each channel over all peptides identified within a file was calculated, and the channel with the highest total abundance was chosen as a reference. After aggregation of all normalized abundance values per sample, the abundance values for each sample were scaled on all averages. Protein ratios were calculated from medians of summed sample abundances of replicate groups. *P*-values were calculated by ANOVA based on individual proteins or peptides. Proteins were determined to be significant if their abundance differential was ≥2-fold (at least log2FC ±1) 23°C and 37°C and had a *P*-value of abundance change *P* < 0.01. The mass spectrometry proteomics data have been deposited to the ProteomeXchange Consortium via the PRIDE partner repository with the data set identifier PXD066216 and null (135).

### Immunoblotting

Western blotting was performed as described previously (136). Protein concentrations were determined by a BCA assay, and equal amounts of total protein (5 μg) of total cellular protein were analyzed by SDS-PAGE and transferred to nitrocellulose. Detection of RpoS was completed utilizing a monoclonal antibody (BioLegend #W0009, clone 1RS1) at a dilution of 1:1,000 and a HRP-conjugated secondary goat anti-mouse IgG (Promega) at a dilution of 1:2,500. Detection of alkaline phosphatase (AP) was completed utilizing a rabbit polyclonal antibody (Thermo Fisher Scientific PA1-26208) at a dilution of 1:15,000 and a HRP-conjugated secondary goat anti-rabbit IgG (Thermo Fisher Scientific) at a dilution of 1:2500. Purified RpoS protein (Neoclone CP009) was used as a positive control. Detection of RpoS and AP was conducted on different gels using the same total protein samples.

## Data Availability

The GEO accession number for the RNA-Seq data reported in this paper is GSE188581. Data for the proteome are available via ProteomeXchange with identifier PXD066216.

## SUPPLEMENTAL MATERIAL

Supplemental tables iai.00422-25-s0001.xlsx Tables S1 to S11.

ASM does not own the copyrights to Supplemental Material that may be linked to, or accessed through, an article. The authors have granted ASM a non-exclusive, world-wide license to publish the Supplemental Material files. Please contact the corresponding author directly for reuse.
